# Cep192, a Novel Missing Link between the Centrosomal Core and Corona in *Dictyostelium* Amoebae

**DOI:** 10.3390/cells10092384

**Published:** 2021-09-10

**Authors:** Valentin Pitzen, Sophia Sander, Otto Baumann, Ralph Gräf, Irene Meyer

**Affiliations:** 1 Department of Cell Biology, University of Potsdam, Karl-Liebknecht-Str. 24-25, 14476 Potsdam-Golm, Germany; valentin.pitzen@uni-potsdam.de (V.P.); s.sander88@gmail.com (S.S.); rgraef@uni-potsdam.de (R.G.); 2 Department of Animal Physiology, University of Potsdam, Karl-Liebknecht-Str. 24-25, 14476 Potsdam-Golm, Germany; obaumann@uni-potsdam.de

**Keywords:** Cep192, SPD-2, centrosome, *Dictyostelium*, microtubule-organization, MTOC

## Abstract

The *Dictyostelium* centrosome is a nucleus-associated body with a diameter of approx. 500 nm. It contains no centrioles but consists of a cylindrical layered core structure surrounded by a microtubule-nucleating corona. At the onset of mitosis, the corona disassembles and the core structure duplicates through growth, splitting, and reorganization of the outer core layers. During the last decades our research group has characterized the majority of the 42 known centrosomal proteins. In this work we focus on the conserved, previously uncharacterized Cep192 protein. We use superresolution expansion microscopy (ExM) to show that Cep192 is a component of the outer core layers. Furthermore, ExM with centrosomal marker proteins nicely mirrored all ultrastructurally known centrosomal substructures. Furthermore, we improved the proximity-dependent biotin identification assay (BioID) by adapting the biotinylase BioID2 for expression in *Dictyostelium* and applying a knock-in strategy for the expression of BioID2-tagged centrosomal fusion proteins. Thus, we were able to identify various centrosomal Cep192 interaction partners, including CDK5RAP2, which was previously allocated to the inner corona structure, and several core components. Studies employing overexpression of GFP-Cep192 as well as depletion of endogenous Cep192 revealed that Cep192 is a key protein for the recruitment of corona components during centrosome biogenesis and is required to maintain a stable corona structure.

## 1. Introduction

Centrosomes are best known for their function as main microtubule organizing centers (MTOCs) [1]. Despite their occasional absence in some subgroups such as higher plants, there is no doubt that they were already part of the inventory of the last eukaryotic common ancestor (LECA) [2,3]. The centrosome is the largest known protein complex of the cell and consists of more than one hundred different proteins (depending on the species) that form several functional groups [4]. It can be distinguished between centriole-containing and acentriolar centrosomes. Centrioles consist of a nine-fold, symmetric, cylindrical assembly of short microtubules. In G1, there is one older centriole, called mother centriole, and one younger centriole, called daughter centriole. Mainly the mother centriole is embedded in a pericentriolar matrix, which contains the majority of the microtubule-nucleation complexes. The centrosome contributes to mitotic spindle assembly and dynamics, and it regulates cytokinesis and cell cycle progression on various levels. It replicates once and only once per cell cycle, ensuring that, after mitosis, mononucleated cells always contain only one single centrosomal entity. In animal cells, the duplication process is regulated primarily by polo-like kinase 4 (Plk4), which organizes the formation of a nine-fold, symmetric, so-called cartwheel structure at the sides of mother and daughter centrioles. The cartwheel then recruits the centriolar microtubules to form the procentrioles [5,6]. The two duplicating centrioles remain connected by interconnecting fibers until the G2/M transition, when the two centriole pairs separate to form the two opposing spindle poles.

Centrioles are structurally related to the basal bodies of cilia and, consequently, centriole-containing centrosomes are found in all organisms capable of forming cilia (i.e., animals and many others). On the other hand, acentriolar centrosomes are typically found in organisms without cilia, including many fungi and amoebozoans [2]. Acentriolar centrosomes have been intensely studied in yeast, where they are called spindle pole bodies, and in the amoebozoan model organism *Dictyostelium discoideum*, where it is also called nucleus-associated body (NAB) [1]. While fungi and animals are in the same eukaryotic supergroup (Opisthokonta), the *Dictyostelium* centrosome provides the best-established model for an acentriolar centrosome outside the Opisthokonta. The *Dictyostelium* centrosome consists of a layered core structure surrounded by a corona, in which γ-tubulin containing nodules are embedded. According to ultrastructural analyses, the core structure consists of three major layers: two outer layers with slightly lower electron density and one central layer with very high electron density [7,8]. A closer look reveals that these major layers can be subdivided into several sublayers. Although earlier work suggested that the layers make up a box-shaped core structure [9], now there is no doubt that they form a cylindrical stack of layers [8,10] as was also found in the related amoebozoan *Polysphondylium violaceum* [11]. Layered structures occur also in yeasts; however, they are most likely analogous to the *Dictyostelium* core layers, not homologous [1].

Unlike in centriolar centrosomes, duplication of the *Dictyostelium* centrosome does not take place during S-phase but starts only at the G2/M transition [12]. First, the whole centrosome increases in size and the corona dissociates, along with the microtubule-nucleation complexes. This is accompanied by the disassembly of all pre-existing microtubules. Next, the remaining core structure enters a fenestra in the nuclear envelope, and the central layer disappears. In prometaphase the remaining outer layers start to separate, each residing in its own fenestra in the nuclear envelope. The former outer layers act as mitotic centrosomes, and upon separation they nucleate spindle microtubules, forming a central spindle. In metaphase, astral microtubules appear. Starting with anaphase, the plaque-shaped mitotic centrosomes undergo a folding process, in which the inner, microtubule-nucleating surface becomes more and more exposed to the cytoplasm. In telophase, the folding process of each mitotic centrosome completes with a scission at the kink of the fold, and the re-appearance of the central layer. This process implicates an inside-to-outside reversal of the outer layers in each cell cycle [12] and implies that the two outer layers have the same protein composition. The new centrosomes then exit their fenestrae in the nuclear envelope, but remain attached to the cytosolic surface of the nucleus. At this time, the microtubule nucleating surface of the new core structure differentiates into the new corona. Our group is still making progress in characterizing the complete set of structural and regulatory proteins involved in this process. Meanwhile, forty-two *Dictyostelium* proteins have been identified as centrosomal or centrosome-associated (reviewed by Gräf et al. in this special issue of Cells). However, despite recent advances in the assignment of novel centrosomal core proteins to the corona and the individual core layers [13,14,15,16,17], the composition of the two outer layers and their interaction with the corona components have remained elusive.

In this paper we focus on Cep192. In mammalian cells, Cep192 (SPD-2 in *Drosophila*) is of special interest in the context of centrosome biogenesis, since it is not only recruited by Plk4 to participate in cartwheel formation and, thus, procentriole formation [18], but is also required to recruit the pericentriolar material around the mother centriole [19]. Here we show that the *Dictyostelium* orthologue of Cep192 is the major component of the outer core layers, and that it interacts with CDK5RAP2, a major recruiting factor of γ-tubulin complexes. In analogy to animal cells, we present evidence that Cep192 is required both for centrosome biogenesis and for integrity of the corona, the functional equivalent of the pericentriolar matrix.

## 2. Materials and Methods

### 2.1. Vector Construction

Exons 1–3, including the complete coding sequence of the *cepH* gene (encoding Cep192), were amplified by PCR using linker primers and subsequently cloned in the N-terminal GFP-fusion vector pIS76 vector to yield the plasmid pIS788 for Blasticidin S selection in *Dictyostelium*.

The Cep192-GFP knock-in plasmid pIS1155 was designed using the empty GFP-knock-in vector pIS1121 according to [17]. In brief, a 642 bp fragment of the Cep192-C-terminal coding region was amplified using linker primers and inserted through KpnI/EcoRI restriction sites adjacent to the GFP sequence. In a second step a 500 bp fragment of the non-coding 3′ untranslated region of the *cepH* gene with added PstI/BamHI restriction sites was cloned into the plasmid downstream of a floxed Blasticidin resistance cassette. The plasmid was linearized by KpnI/BamHI digestion prior to transformation into *Dictyostelium* AX2 cells. The two inserted fragments act as polylinkers in the following transformation and promote homologous recombination resulting in the replacement of Cep192 by Cep192-GFP. As all recombination events take place downstream of the promoter, the encoded fusion protein is expressed under the control of the endogenous *cepH* promoter.

The Cep192-RNAi construct pIS701 was prepared as described in [20]. A sense strand consisting of base positions 1–453 of the *cepH* coding sequence was amplified using SalI/SacI linker primers along with the corresponding, reverse complement fragment flanked by AflII/KpnI restriction sites. Both fragments were cloned into the pIS193 vector adjacent to each side of a short spacer sequence originally derived from mCherry. The plasmid was transformed into AX2 cells.

The Cep192-SpotH6 plasmid (pIS1314) was made using the sequence of the Spot-tag (Chromotek, Planegg-Wiedmannsried, Germany [21]) together with a 6×His-tag flanked by EcoRI/HindIII restriction sites, replacing the GFP tag in the aforementioned Cep192-GFP knock-in plasmid pIS1155. The 6×His-tag (H6) was added to the Spot-tag to facilitate potential purification approaches.

The BioH6-tag is a codon-optimized version of the 75 amino acid Bio-tag described in [22,23]. The codon-optimized sequence was obtained by gene synthesis (GeneArt, Thermo Fisher Scientific, Waltham, MA, USA) and subsequently the BioH6-tag was used to replace the tags in different available knock-in plasmids for centrosomal proteins (made as described in detail for pIS1155), yielding the plasmids pIS1352 (Cep192-BioH6), pIS1361 (CDK5RAP2-BioH6), pIS1362 (CP39-BioH6), and pIS1378 (CP91-BioH6). The BioH6-tag exhibited effective biotinylation of the tagged proteins at the default biotin concentrations provided by HL5c medium (Formedium, Hunsanton, UK). The plasmids were transformed into IS584 cells already containing the SpotH6-tagged Cep192 construct, or AX2 cells. Strain IS584 resulted from an excision of the floxed blasticidin cassette by transient transformation of a Cre-recombinase encoding plasmid [24] into the Cep192-SpotH6 knock-in strain. The cells were then screened for loss of the Blasticidin resistance, yielding the resistance-free strain IS584. Therefore, the Blasticidin resistance could be re-used to introduce further knock-in constructs.

The same strategy was applied to generate the other knock-in plasmids. Two BioID2 knock-in constructs were produced. The sequence of the BioID2-tag was obtained from the pIS1199 knock-in plasmid and cloned via HindIII/EcoRI into pIS1164 (CDK5RAP2-GFP) and pIS1155 to yield pIS1251 (CDK5RAP2-BioID2) and pIS1263 (Cep192-BioID2). For the FLAG-BioID2 control strain, the coding sequence of BioID2 was inserted into a FLAG-tag containing derivative of the pIS76 plasmid resulting in pIS1382 for Blasticidin S selection in *Dictyostelium*.

### 2.2. Fluorescence Microscopy

Cells were fixed with methanol at −20 °C for 3 min, or with glutaraldehyde as described in [25].

Wide field fluorescence microscopy was performed as described previously [26], using a AxioObserver system equipped with a Plan-Apochromat 100×/1.4 oil immersion objective, a LED light source (Colibri7, Carl Zeiss Mikroskopie GmbH, Jena, Germany), and an AxioCam506 mono or a Zeiss Axiovert 200M system with Zeiss HXP120 lamp using a Plan-Apochromat 100×/1.4 oil immersion lens and an AxioCam MR3 (Carl Zeiss Mikroskopie GmbH, Jena, Germany).

Live cell imaging was performed with a Cellobserver SD confocal spinning disk system equipped with an LCI-Plan-Neofluar 63×/1.3 lens (Carl Zeiss Mikroskopie GmbH, Jena, Germany) and an Evolve EM-CCD camera (Photometrics, Tucson, AZ, USA). Cells were allowed to settle, the medium was changed to LoFlo (Formedium, Hunsanton, UK), and 2 mg/mL ascorbic acid were added to reduce phototoxic effects. If necessary, cells were flattened by agar overlay [27]. For FRAP analysis, the 473 nm laserline of a Rapp UGA-40-2L Galvo scanner (Rapp Optoelectronics, Hamburg, Germany) was used for bleaching. Analysis of FRAP data was performed according to [28]. Expansion microscopy was performed based on [29,30] as outlined in [31]. In this study, to achieve maximal staining, two-fold higher concentrations of the conjugates were used and the incubation was carried out overnight at 4 °C. Expanded specimens were viewed on a LSM880 with Airyscan detector equipped with a Plan-Apochromat 40×/1.2 water immersion objective (Carl Zeiss Mikroskopie GmbH, Jena, Germany).

### 2.3. Electron Microscopy

Cells were fixed as described previously [16] and flat-embedded in Agar Low Viscosity medium (Plano GmbH, Wetzlar; [25]). Ultrathin sections (80–90 nm) were stained with uranyl acetate and lead citrate and analyzed in a Talos F200C TEM (Thermo Fisher Scientific, Waltham, MA, USA), operated at 200 keV.

### 2.4. Antibodies and Conjugates

Primary antibodies used in this study: anti-Cep192 [17], anti-CP39 [13], anti-CP55 [14], anti-CP91 [15], anti-CP148 [16], anti-CDK5RAP2 [17], anti-CP224 [32], anti-NE81 [33], anti-α-tubulin [34]. Secondary antibodies and fluorescent Streptavidin were purchased from Thermo Fisher Scientific (Darmstadt, Germany), the Spot-Label nanobody recognizing the SpotH6-tag was obtained from Chromotek (Planegg-Wiedmannsried, Germany), and enzyme conjugates for Western blotting were from Sigma (Deisenhofen, Germany).

### 2.5. Cell Culture

Cells were cultured in HL5c medium (Formedium, Hunstanton, UK) with sterile filtered glucose added after autoclaving, and 10 µg/mL G418 or 4 µg/mL Blasticidin S, if needed. For microscopy, cells were grown in adherent culture using tissue culture flasks, and for centrosome isolations [35] and BioID2 analyses, shaking culture was used as described previously [36].

### 2.6. Other Methods

Standard protocols were applied for SDS electrophoresis, Western blotting, and transformation of *Dictyostelium* amoebae by electroporation. BioID2 analyses were largely performed as described earlier [13,37], however the biotin concentration was reduced to 2 µM acknowledging the higher biotinylation efficiency of the BioID2 biotinylase [38]. In short, centrosomes were isolated from the corresponding strains and loaded on Western blots. Broad single Western blot lanes were split in stripes, stained individually with streptavidin-alkaline phosphatase or antibodies as indicated on top, and were re-aligned afterwards. Bands were visualized using antibody conjugates with alkaline phosphatase and NBT/BCIP color detection.

## 3. Results

Cep192 was among the novel components of the *Dictyostelium* centrosome identified by our proteomic analysis of isolated centrosomes [39]. In an early survey of the light microscopic localization of GFP-fusion proteins derived from all centrosomal candidates found in this proteomic approach, we were unsure whether Cep192 should be designated to the core structure, or to a previously uncharacterized inner part of the corona directly attached to the core layers [40]. A close association with the core structure appeared likely also due to the presence of Cep192 at metaphase spindle poles [40]. Yet, we could not exclude that the latter was a result of GFP-Cep192 overexpression, and that Cep192 localization within the densely packed centrosomal proteins was affected by the bulky GFP-tag. In this work we clarify these issues and show that Cep192 is an essential part of the outer layers of the centrosomal core structure and is required for the integrity of the microtubule-nucleating corona.

**Figure 1:**
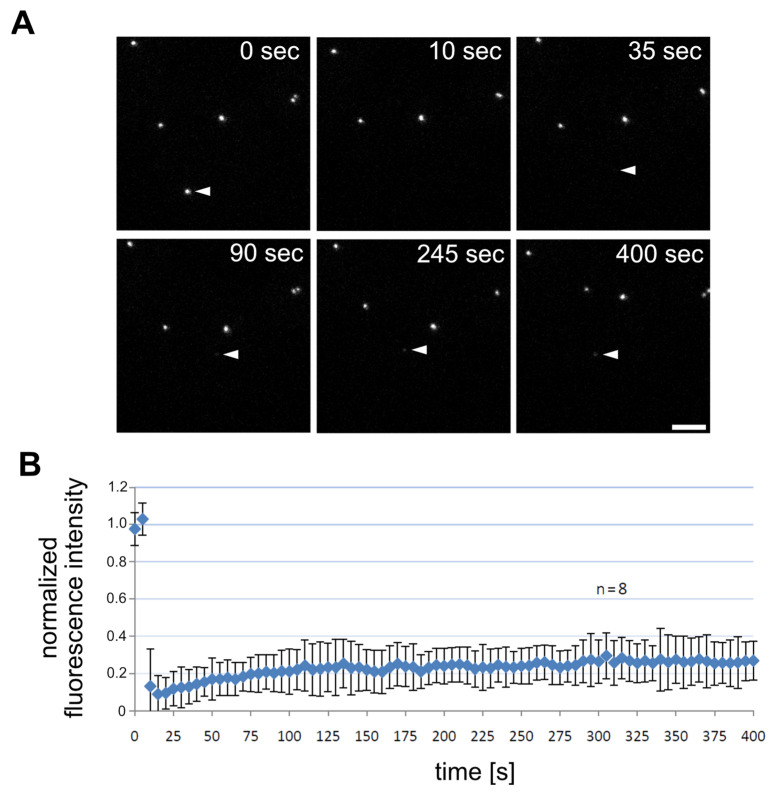
GFP-Cep192 shows hardly any recovery after photobleaching. (**A**) Selected timepoints of a photobleaching experiment (Video S1), Bar = 5 µm. Photobleaching was performed at the filled arrowhead. (**B**) Evaluation of fluorescence recovery of eight individual GFP-Cep192 bleachings.

To confirm that Cep192 is a structural centrosomal component we first performed FRAP experiments with our GFP-Cep192 strain, which expresses the fusion protein in addition to the endogenous Cep192. [40]. Unlike corona components involved in microtubule nucleation and dynamics such as CP224 (XMAP215 orthologue), which show rapid recovery at the centrosome after photobleaching during interphase (t^1^/_2_ = 7.2 s; [41]), GFP-Cep192 showed hardly any recovery after 400 s (Figure 1), which is typical for centrosomal core components and scaffolding proteins of the corona [14,15,16,17].

**Figure 2:**
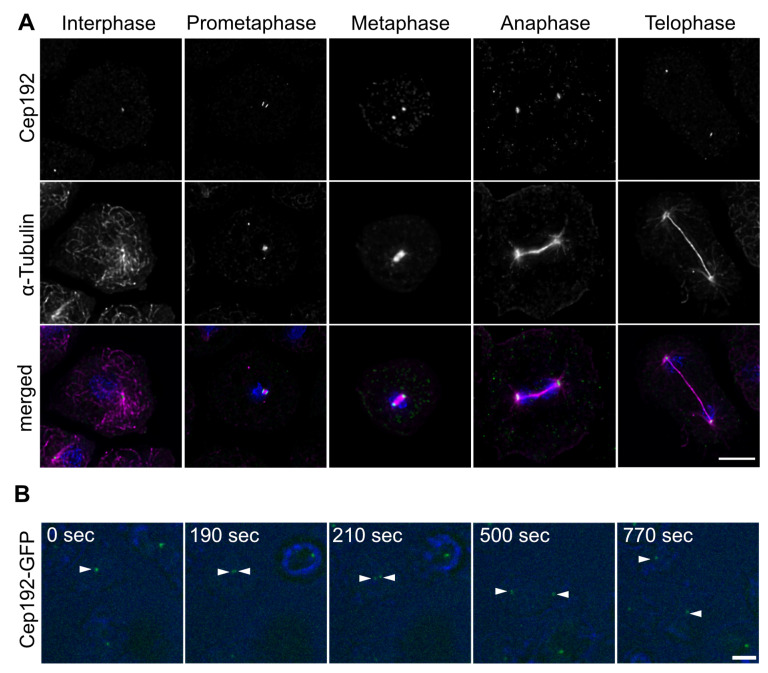
Cep192 is present at the mitotic spindle poles. (**A**) Immunofluorescence microscopy of AX2 cells in interphase and indicated mitotic stages, stained with anti-Cep192 and anti-α-Tubulin. Secondary antibodies were anti-rabbit-AlexaFluor-488 and anti-rat-AlexaFluor-568, cells were fixed with methanol, DNA stained with DAPI. (**B**) Cep192-GFP is present during the splitting of the mitotic centrosome. Selected time points from Video S2 are displayed. Cells were viewed under agar overlay. Bars = 5 µm.

Yet, these data did not clarify whether Cep192 is a structural scaffold component of the corona, or the layered core. A further argument for the latter came from studies of the localization of endogenous Cep192 during mitosis. In samples stained with our anti-Cep192 antibody, widefield deconvolution microscopy revealed an association of endogenous Cep192 with centrosomes, both during interphase and throughout all mitotic stages (Figure 2A).

As the central layer of the core structure disappears in prophase and re-appears only in telophase, this is in line with the idea that Cep192 could be a part of the outer core layers. In contrast to our earlier study, we can now exclude that mitotic centrosomal localization was due to an overexpression effect as, e.g., in case of the highly overexpressed central layer component GFP-CP75 [42]. The mitotic behavior of Cep192 was confirmed in live cells carrying a knock-in of Cep192-GFP. In these cells the fusion protein is expressed under the control of the Cep192 promoter, replacing the endogenous protein. Video S2 clearly shows that Cep192-GFP remains located at the centrosome from the G2/M transition until the next interphase (Figure 2B).

Although these data strongly suggested that Cep192 is a constituent of the outer core layers, we still had no direct microscopic proof of this notion. Due to the small size of the core structure (diameter ≈280 nm, thickness ≈140 nm; [10,14,43]; see also EM analysis in this work) conventional light microscopy with deconvolution (resolution limit with our system 170 nm [17]) or Airyscan confocal microscopy provided insufficient resolution to solve this problem. Therefore, we decided to employ expansion microscopy (ExM), a meanwhile established superresolution method, based on the unprecedented quality of animal centriole imaging by ExM in the Guichard lab [44] as well as our own positive outcome in the study of *Dictyostelium* lamin [31].

**Figure 3:**
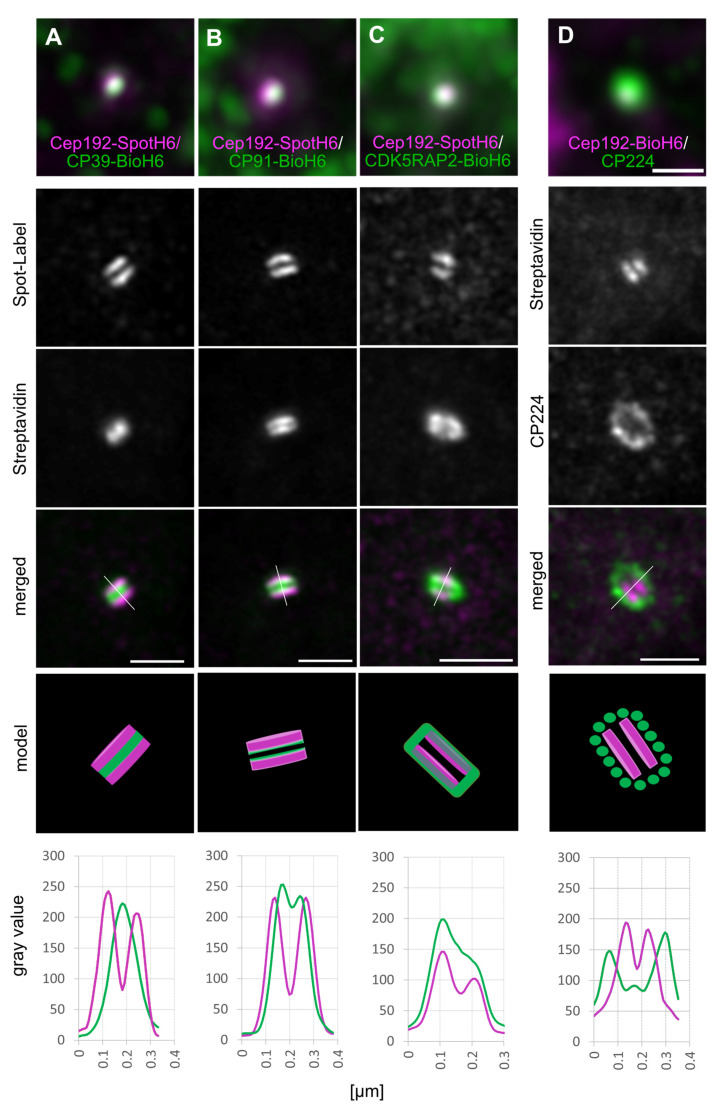
Subcentrosomal distribution of CP39-BioH6 (**A**), CP91-BioH6 (**B**), CDK5RAP2-BioH6 (**C**), and CP224 (**D**) relative to Cep192-SpotH6 (**A**–**C**) and Cep192-BioH6 (**D**). First row represents merged images of unexpanded specimen, gray scale images and corresponding merged images are expanded specimen, resulting in the localization models and intensity distribution graphs along the drawn lines in the merged images. Cells were fixed with methanol and stained with anti-Spot-Atto-594 nanobody (**A**–**C**), Streptavidin-AlexaFluor-488 (**A**–**D**), and anti-CP224/anti-mouse-568 (**D**). Shown are maximum intensity projections of Airyscan processed (expanded) or deconvolved images (non-expanded). Scale bar = 500 nm, for the expanded specimen the scale was fitted using the expansion factor to represent the original size of the structure.

In all superresolution light microscopy techniques using couples of primary and secondary, fluorescently labeled antibodies should be avoided since the distance of the fluorophore from the antigenic epitope may extend up to 30 nm [45]. To minimize the distance between epitope and fluorophore, we tagged Cep192 and suitable centrosomal reference proteins with short tags for which probes smaller than primary and secondary antibodies were available. For Cep192 we decided on the SpotH6-tag and corresponding anti-Spot nanobody [21]. To avoid overexpression effects, we created a *Dictyostelium* Cep192-SpotH6 knock-in strain using the same homologous recombination technique as for the Cep192-GFP strain mentioned above and re-transformed it with further knock-in constructs. We used CP39 as a centrosomal reference protein for the central layer of the core structure [13]. To allow later staining with fluorescent streptavidin, we tagged the C-terminus of CP39 with a biotinylation sequence and a 6×His-tag (together called BioH6-tag). When expressed in *Dictyostelium* cells the BioH6-tag of the fusion protein becomes biotinylated by endogenous biotinylases. Cep192-SpotH6 cells were subsequently transformed with the CP39-BioH6 knock-in construct to yield the Cep192-SpotH6/CP39-BioH6 strain, in which both endogenous proteins were replaced by the respective fusion protein expressed under control of the respective endogenous promoters. These cells were then used for ExM using the anti-Spot-Atto594 nanobody and AlexaFluor488-labeled streptavidin to visualize the respective tags. ExM images clearly showed that Cep192 is present in two discrete layers and that CP39 is localized exactly in between these layers (Figure 3A). This localization, the size, and the appearance of the whole labeled structure perfectly support our hypothesis that Cep192 is a constituent of the outer core layers, and CP39 is a constituent of the central core layer. Moreover, distances of the Cep192-SpotH6 fluorescence peaks along straight line selections through both layers (110 ± 15 nm, mean ± SD, n = 25) and the diameter of the cylindrical core structure (FWHM = 231 ± 23 nm, mean ± SD, n = 6) perfectly matched the size of the core structure in electron microscopic images of control cells (diameter ≈284 ± 42 nm, thickness ≈142 ± 18 nm (mean ± SD, n = 25); Figure 7).

As a further reference protein for the central layer, we used CP91 and transformed a CP91-BioH6 knock-in construct into Cep192-SpotH6 cells to yield the Cep192-SpotH6/CP91-BioH6 strain. ExM revealed a distribution of CP91 in two adjacent but distinct layers, which were flanked at their distal ends by Cep192 (Figure 3B). This demonstrates the existence of more than three layers within the core structure, confirming early electron microscopic data [8,9].

In order to investigate Cep192-SpotH6 localization in relation to a bona fide corona component, we created a further knock-in strain by transformation of CDK5RAP2-BioH6 into our Cep192-SpotH6 strain. Again, the resulting CDK5RAP2-BioH6/Cep192-SpotH6 cells no longer expressed the respective endogenous proteins, only the tagged versions. ExM revealed that CDK5RAP2 was arranged in a ring-like pattern partially overlapping with Cep192, suggesting a close association of both proteins (Figure 3C). This pattern was not surprising since our earlier analysis using deconvolution fluorescence microscopy had already shown that, within the corona, CDK5RAP2 is localized more proximally to the core structure than the other corona marker component CP224 [17]. Our attempts to create a corresponding knock-in strain carrying CP224-BioH6 instead of CDK5RAP2-BioH6 have been unsuccessful. Therefore, we used the anti-CP224 monoclonal antibody and secondary anti-mouse-AlexaFluor568 to label CP224 for ExM. For this we created a Cep192-BioH6 knock-in strain in the same manner as the Cep192-SpotH6 strain. As expected, Cep192-SpotH6 was clearly localized within the CP224 labeled corona, despite the lower resolution of anti-CP224 staining (Figure 3D). Moreover, both sizes and shapes of the structures visualized using either of the two tags BioH6 or SpotH6 were practically identical, also with regard to labeling intensity and resolution (Figure 3D).

**Figure 4:**
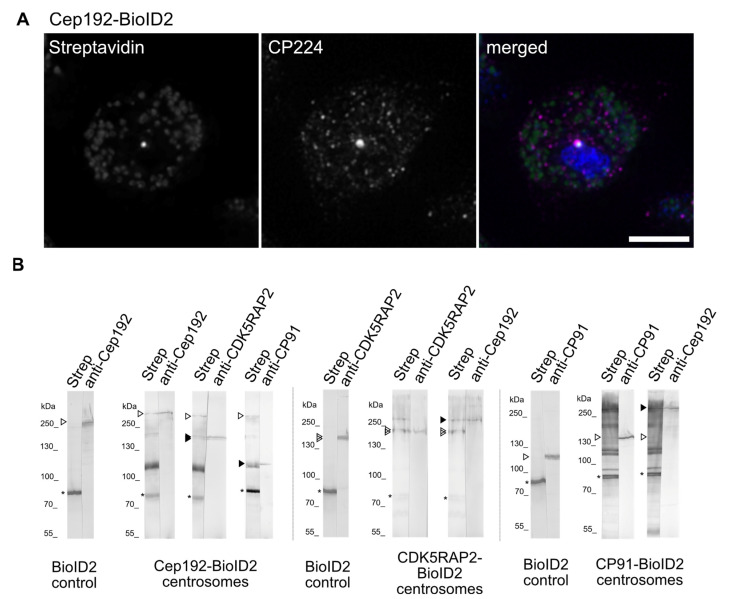
Centrosomal interactions of C-terminally biotinylase-tagged Cep192, CDK5RAP2, and CP91 in the BioID2 assay. (**A**) Immunofluorescence microscopy of methanol-fixed Cep192-BioID2 cells after treatment with 2 µM Biotin stained with anti-CP224/anti-mouse-AlexaFluor-568 and streptavidin-AlexaFluor-488, Bar = 5 µm. (**B**) BioID2 Western blot analysis of centrosomal fractions of Cep192-BioID2, CDK5RAP2-BioID2, and CP91-BioID2 cells. The nitrocellulose membrane was cut into lanes, whereby each lane was cut into equal halves. After blotting, half-lanes were stained with the indicated antibodies and streptavidin, respectively. ‘Strep’ refers to the individual biotinylation pattern detected by alkaline phosphatase coupled Streptavidin. Open arrowheads represent the fusion proteins and the respective endogenous proteins in the control cells, the size difference is due to the 27kDa BioID2 tag. Filled arrow heads highlight potential interactors found by co-staining with their respective antibody. As secondary antibody anti-rabbit-alkaline-phosphatase was used. Bands were visualized using NBT/BCIP color detection. Controls expressed FLAG-BioID2 alone and were treated the same way. Stars indicate endogenously biotinylated proteins always visible in streptavidin staining.

The partial overlap of CP91 and Cep192 on the one hand and CDK5RAP2 and Cep192 on the other hand suggested at least a close proximity, or even a direct interaction of Cep192 with these two proteins. To address this question more closely, we decided to employ BioID. Here a protein of interest is fused to a promiscuous biotinylase, which biotinylates lysine residues within a proximity of up to 10 nm [46]. Thus, after streptavidin affinity isolation of biotinylated target proteins, predominantly direct interactors can be identified by mass spectrometry or Western blotting. Meanwhile BioID has turned out to be the most effective method to determine the centrosomal interactome, in our lab [13,17] and also in others [47,48,49]. Compared to co-precipitation assays it does not require solubility of the interaction partners. A further difficulty of co-precipitation assays with centrosomal proteins is that they are often virtually absent from soluble cell extracts and can only be solubilized after dissociation of isolated centrosomes, whereby various artificial subcomplexes are generated (own observations). By contrast, the BioID assay reliably detects close proximity under in vivo conditions. For the biotinylase tag we selected BioID2 based on its smaller size and enhanced labeling of adjacent proteins compared to the classical BirA-R118G (BirA*) [38]. To avoid overexpression of the BioID2-fusion proteins we employed again our knock-in strategy (see above). Fluorescence microscopy revealed biotinylation of centrosomal targets by Cep192-BioID2 (Figure 4A). For the assay we loaded isolated nucleus/centrosome complexes of the respective BioID2 strains on broad SDS page lines and cut the corresponding western blot membrane in stripes. Adjacent stripes were then stained with streptavidin-alkaline phosphatase and centrosome-specific antibodies in parallel (Figure 4B).

By applying this procedure we were able to correlate the biotinylated bands with our known centrosomal proteins. Even without the prior isolation of the biotinylated proteins, by using highly enriched protein samples this correlation should result in the reliable identification of proteins of high proximity, and therefore very likely interaction partners. Alongside with biotinylated target proteins, the endogenously biotinylated mitochondrial methylcrotonyl-CoA carboxylase alpha (MccA) (77 kDa), propionyl-CoA carboxylase alpha (PccA) (80 kDa), and acetyl-CoA carboxylase (AccA) (257 kDa, often weak band) are always visible in streptavidin-stained Western blots [37]. These are also the only bands visible in our negative control with a strain expressing BioID2 alone (Figure 4B).

Using this method, we identified CDK5RAP2 as a likely biotinylation target for Cep192-BioID2. A band corresponding to the size of CP91 was labeled with streptavidin as well; however, it appeared thicker than the CP91 band on the same blot, suggesting that there could be more than one biotinylated protein of about this size which we will try to identify with mass spectrometry in future studies. Vice versa, Cep192 was biotinylated by both CDK5RAP2-BioID2 and CP91-BioID2 in the corresponding knock-in strains. Taken together, these BioID results are in agreement with the topology and association of CDK5RAP2, Cep192, and CP91 detected by ExM, indicating a very likely direct mutual interaction of these proteins. Cep192-BioID2 did not biotinylate any other known corona or core components we tested for with our antibodies. The tight association of Cep192 and CDK5RAP2 is also supported by an observation in *Dictyostelium* cells overexpressing GFP-CDK5RAP2. These cells frequently displayed cytosolic and nuclear clusters formed by the fusion protein. While the cytosolic clusters contained both corona and core proteins, the nuclear clusters additionally contained the core proteins CP55 and CP91 [17]. Cep192 also robustly colocalized with these nuclear clusters (Supplementary Figure S1) and, thus, it mimicked the behavior of the other core components.

Taken together, our BioID and ExM data illustrate a centrosomal topology, in which Cep192 makes up the outer core layers flanking the central layer. The contact surfaces of the central layer consist of CP91, and its inner zone contains CP39. CP75, which interacts with both CP39 and CP91, could not exactly be assigned within the central layer in this work [13]. On their outer surface, the outer core layers interact mainly with CDK5RAP2, which in turn is involved in organizing the corona consisting of γ-tubulin complexes, CP148, CP224, and CP248/250 [17].

**Figure 5:**
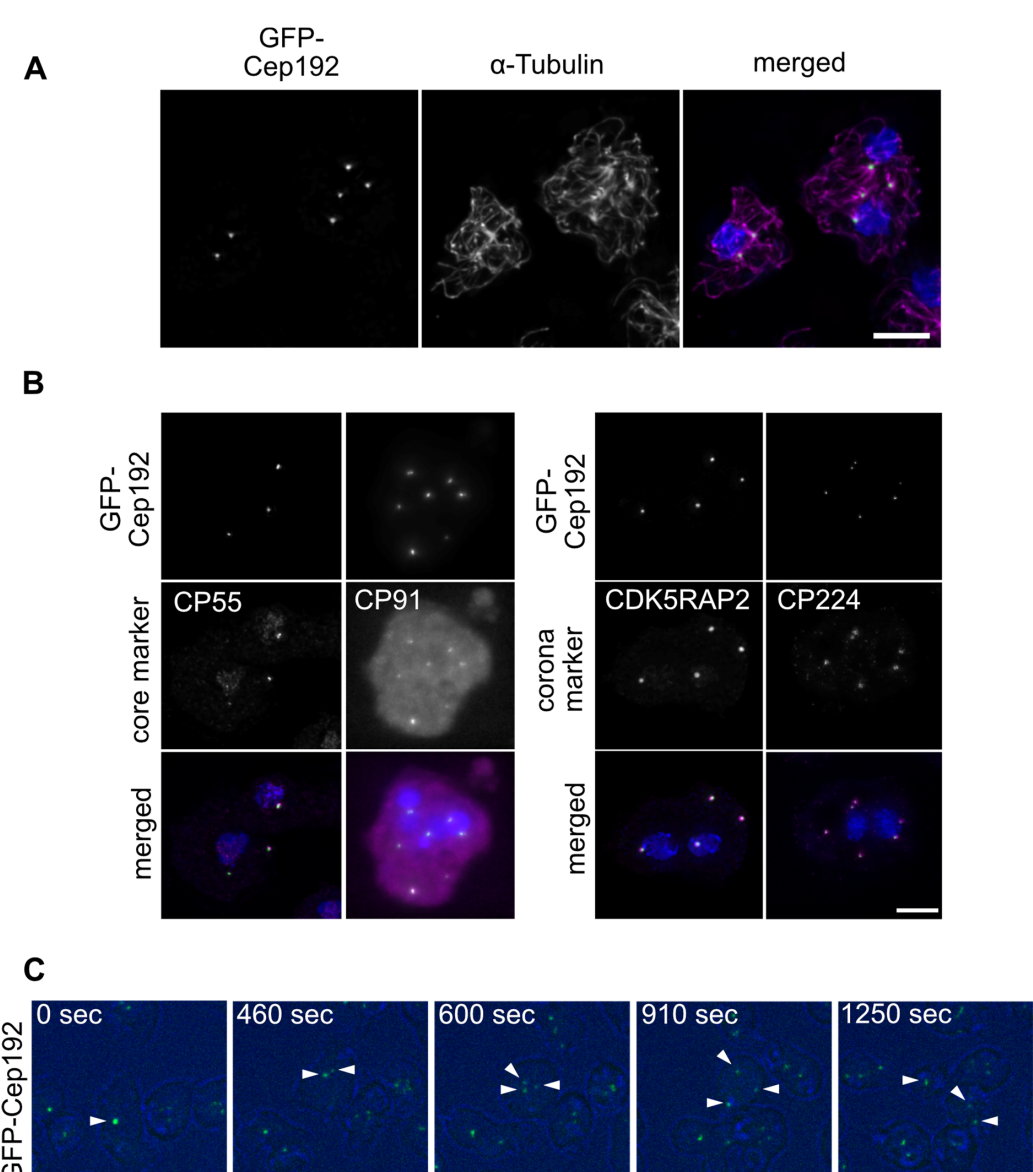
Immunofluorescence microscopy of GFP-Cep192 overexpression elicits supernumerary MTOCs. (**A**) Additional GFP-Cep192 foci co-stained with anti-α-tubulin/anti-rat-AlexaFluor-568 revealing the MTOC property. (**B**) Co-staining of different core and corona marker proteins with indicated antibodies. Secondary antibody was anti-rabbit-Alexa-Fluor568. Cells were fixed with methanol, shown are deconvolved maximum intensity projections. (**C**) Live cell imaging of GFP-Cep192 during mitosis. Selected time points from Video S3 are shown. Arrowheads point at emerging GFP-Cep192 foci. Bar = 5 µm.

To further assess the functional role of Cep192 we created overexpression and depletion strains. Overexpression of GFP-Cep192 elicited cytosolic supernumerary MTOCs in 78% (n = 448) of all cells (Figure 5A). Co-staining of these cells with specific antibodies revealed the presence of CP55 and CP91 as representatives of the outer and central core layers, respectively (Figure 5B), strongly suggesting that these supernumerary MTOCs represent complete centrosomes as also observed in other cell lines overexpressing centrosomal components [50]. Observation of live cells revealed that supernumerary centrosomes arise during mitosis (Figure 5C, Video S3). The centrosomal aberration caused by GFP-Cep192 overexpression support a role of Cep192 as an early recruiter of further centrosomal components during mitotic centrosome biogenesis.

**Figure 6:**
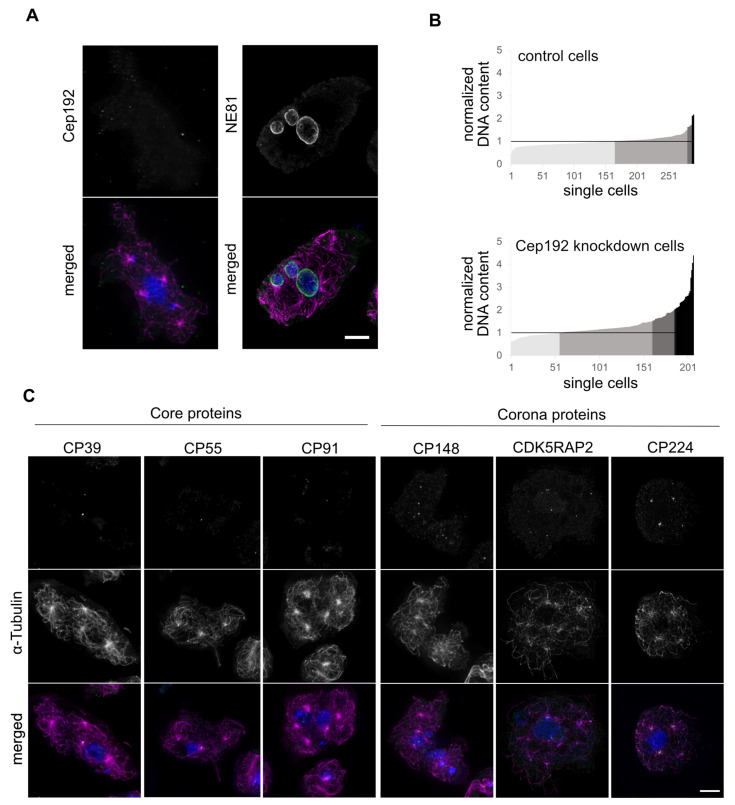
Cep192-RNAi results in supernumerary MTOCs and increased DNA content. (**A**) Cells with supernumerary MTOCs stained with anti-Cep192 or anti-NE81 and anti-α-Tubulin (magenta). (**B**) Comparison of DNA content of control cells expressing GFP-α-Tubulin [51] with Cep192-RNAi cells. Cells were mixed for immunofluorescence microscopy and were distinguishable by GFP fluorescence. DNA content is determined by the product of intensity (16-bit gray scale) and area (µm^2^) of the DAPI staining for n = 290 control cells and n = 207 Cep192-RNAi cells. The mean control value of the product was set to one for normalization. Increase in DNA content is shown in the graphs and indicated by darkening gray scale: darker gray indicates cells with 1.5-fold higher, black over twofold higher, DNA content. (**C**) Immunofluorescence microscopy of Cep192-RNAi cells with α-Tubulin and different stainings for indicated core and corona proteins. Cells in A and C were fixed with methanol and secondary antibody was anti-rabbit-AlexaFluor-488 and anti-rat-Alexa-Fluor-568. DAPI was used for DNA staining in the merged images. Maximum intensity projections of deconvolved images are shown. Bar = 5 µm.

**Figure 7:**
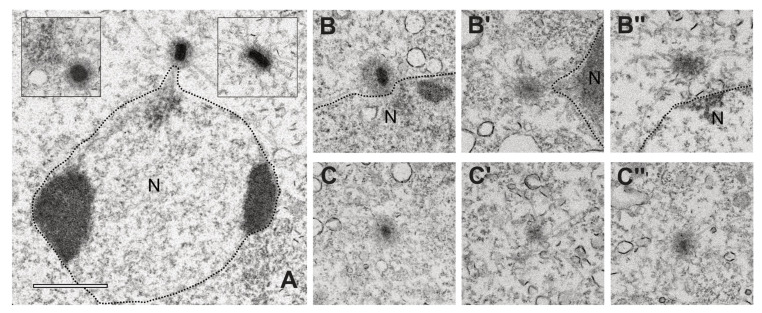
TEM images of centrosomes and MTOC. Centrosomes of AX2 control cells (**A**, with insets) and Cep192-RNAi centrosomes (**B**–**B’’**) and MTOCs (**C**–**C’’**). The image of the control centrosome shows the attachment of the centrosome at the nucleus (N), the two insets show two different orientations of the core structure, with a view on top (left) and from the side (right). The cells were fixed with glutaraldehyde and after embedding sliced and imaged as 80–90 nm thick slices. Bar = 1 µm.

Next, we wanted to study the phenotype resulting from depletion of Cep192. Even though a knock out of CP55 was possible, all three other core proteins turned out to be essential [13,14] and we assumed that Cep192 would be essential as well. Therefore, we chose to deplete Cep192 by RNAi using the method of Martens and co-workers [20]. To evaluate the extent of Cep192 depletion we mixed Cep192RNAi cells with equal amounts of GFP-α-tubulin cells and stained with anti-Cep192 and an AlexaFluor 568 conjugated secondary antibody (Figure S2). Cells with green microtubules were used as an internal reference for normal Cep192 levels in these specimens, whereas cells with unstained microtubules represented the Cep192RNAi cells. The latter showed a reduction of Cep192 staining intensity by ~28.5% (SD = 9.9%, n = 56). When analyzing for centrosomal phenotypes, again we observed supernumerary MTOCs in ~39% (n = 227/585) of all cells (Figure 6A). In contrast to supernumerary MTOCs elicited by Cep192 overexpression, these supernumerary MTOCs were virtually devoid of detectable core layer components, (Figure 6C), while both contained CDK5RAP2, CP148, and CP224. Thus, supernumerary MTOCs elicited by Cep192 knockdown are just MTOCs, not bona fide centrosomes. This was confirmed by electron microscopy of Cep192RNAi cells (Figure 7). Supernumerary, cytosolic MTOCs were devoid of a clearly discernible layered core structure. At best, they showed an increased electron density in their center. In many cases, nucleus-attached MTOCs also lacked a clearly discernible, layered core structure. The nucleus-attached centrosomes were affected by Cep192 depletion as well, the outer layers showed a lower electron density and often had a smaller diameter than the central layer.

Overall, the situation is reminiscent of that observed in CP55 knockout cells, which were also characterized by supernumerary MTOCs but not centrosomes [14]. As another similarity to CP55 knockout cells we also observed a mild increase in ploidy in the Cep192-RNAi strain. The average DNA content was increased more than 1.5-fold in ~23% (n = 47/207, control ~3% n = 10/290) of all cells (Figure 6C). On the microscopical level this was reflected by variable nuclear sizes, i.e., the appearance of both mini-nuclei and unusually large nuclei (Figure 6A).

## 4. Discussion

In this work we show that Cep192 is a major, if not the most important, structural component of the outer core layers of the *Dictyostelium* centrosome. This view is based on the fact that neither our own centrosomal proteome analysis nor our BioID studies (this study and [13,17,39]) have revealed further candidate proteins for the outer core layers, in addition to Cep192, Nek2, and the non-essential CP55. In fact, CP55 is the only structural centrosomal component so far that was successfully knocked out in *Dictyostelium* [14]. For all further proteins tested, RNAi depletion already caused severe growth defects. Nek2 is mentioned here since it was the first centrosomal core component identified as a permanent centrosomal resident [52]. Whether this NIMA-related kinase plays a structural role at the centrosome is unknown.

**Figure 8:**
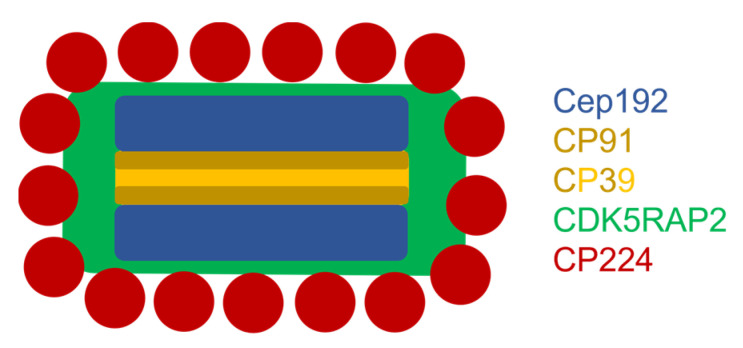
Schematic drawing of the topology of the investigated proteins at the *Dictyostelium* centrosome. Depicted is a cross section through the centrosome: CP91 resides in the inner core layer as two distinguishable layers (darker yellow) and in close proximity to Cep192 (blue), which is located in the outer core layers. CP39 (yellow) also locates in the inner core layer. CDK5RAP2 (green) is a protein closely associated with the core structure and locates in the corona together with CP224 (red).

Superresolution expansion microscopy clearly revealed Cep192 at the outer core layers of the centrosome. The core structure is coated by CDK5RAP2 and more distantly surrounded by other corona proteins including CP224 (Figure 8). Furthermore, for the first time our ExM images disclosed the existence of more than three layers in the core structure on the light microscopical level, since CP91 was localized in two distinct inner layers flanked by the Cep192 layers. However, from a functional point of view it still makes sense to speak of three major core layers, i.e., two outer and one central layer. On the ultrastructural level only the two outer layers remain during mitosis, while the central layer including the CP39, CP75, and CP91 components disappears during mitosis.

Gambarotto and co-workers have shown that classical ExM employing pre-expansion staining with primary and secondary antibodies could lead to misinterpretations of dimensions when applied to dense structures, such as centrioles, that are accessible for antibodies predominantly on their outer surface [44]. Thus, they introduced an improved method called U-ExM (U stands for ultrastructure), employing post-expansion staining and a modified cross-linking protocol. Yet, in this study we show that a combination of the classical pre-expansion staining ExM method with the use of small tags in combination with small fluorescent probes also eliminates the known problem of misinterpreted dimensions observed with classical ExM. The dimensions of our labeled Cep192 structures nicely fit the dimensions, both in terms of size and distance, of the outer core layers deduced from electron microscopic images.

Cep192 belongs to those centrosomal proteins that are capable of oligomerization [18], and this is crucial for Cep192 to serve as a scaffolding protein, together with pericentrin and CDK5RAP2, in the organization of the pericentriolar matrix [53,54,55,56]. This scaffold recruits and binds γ-tubulin complexes in an Aurora A and Polo-like kinase 1-dependent manner [57,58].

Orthologues of CDK5RAP2 (Cnn/Cep215/Spc72p) and pericentrin (Kendrin/PLP/Spc110p) are found in organisms with centriole-containing and with acentriolar centrosome types, such as animals and yeast. In *Dictyostelium* these proteins are represented by CDK5RAP2 (also named Cep161 in *Dictyostelium*; [17,59]) and CP148. Despite low sequence similarity, the latter is considered the pericentrin homologue, as it behaves in a functionally similar manner to pericentrin and also contains the typical IQ-domains and long coiled-coil regions [16]. Among these proteins, Cep192 has been so far found only in animals and amoebozoa (*Dictyostelium*). With its corona the *Dictyostelium* centrosome contains a structure highly reminiscent of the pericentriolar matrix, in contrast to yeasts. This prompted the hypothesis that PCM recruitment could be the ancestral function of Cep192 [60]. In other words, Cep192 seems to have evolved with the capability to form a microtubule-nucleating matrix around the core replicatory unit (i.e., centrioles or layered core structure). Indeed, overexpression of GFP-Cep192 in mammalian cells resulted in the formation of multiple GFP-Cep192 foci, also including γ-tubulin and pericentrin, i.e., two major components of the PCM [61]. This is in line with our observations in *Dictyostelium*, where cytosolic GFP-Cep192 foci also contained further corona components (Figure 5). However, compared to mammalian cells, the assembly of these foci even went further in *Dictyostelium.* They actually behaved like MTOCs or even centrosomes, as they were able to nucleate microtubules and to recruit centrosomal core components. Despite these differences, this strongly supports a major, conserved role of Cep192 in the recruitment of PCM and its functional equivalents. The failure of overexpressed GFP-Cep192 to induce de novo assembly of complete centrosomes in mammalian cells may be due to the inability of Cep192 alone to recruit the essential initiators of cartwheel/procentriole assembly, e.g., Plk4 or Cep152 [62,63,64]. These proteins appear to be absent from the *Dictyostelium* genome. Thus, Cep192 seeds that could result from oligomerization of the overexpressed protein could be sufficient to recruit further centrosomal proteins to assemble complete, supernumerary centrosomes. Moreover, if Cep192 is the main component of the outer layers, and the outer layers are practically all that is left of the centrosome after splitting of the core structure in the early duplication phase, it is not far-fetched that cytosolic Cep192 foci could serve as seeds for the formation of complete centrosomes in the same way as the mitotic outer layers do naturally.

A role of Cep192 in the recruitment of PCM-like material, i.e., the corona, is also supported by the phenotype of the Cep192 knockdown strain. In mammalian cells, optimized siRNA-mediated knockdown of Cep192, which resulted in a 90% reduction of the protein at the centrosomes, leads to a strong reduction of microtubule nucleation at the centrosome [65]. For knockdown approaches in *Dictyostelium* cells we used stably integrated RNAi constructs and selected for viable strains, which, in case of essential proteins, still express sufficient protein for the strains to survive. The resulting Cep192 knockdown cells showed only a 30% reduction of Cep192 at the centrosomes and were characterized by supernumerary, cytosolic MTOCs. In addition, they often contained ultrastructurally abnormal, nucleus-associated centrosomes with reduced outer layers or crippled core structures. Our interpretation is that Cep192 depletion destabilizes the corona, and the pulling and pushing forces exerted by microtubules [66] then result in the detachment of fragments from the corona, which could then act as supernumerary MTOCs. At the onset of mitosis they shed their microtubules and disassemble in the same manner as the centrosomal corona. In telophase they may re-form at remaining seeds containing corona material. During a semi-closed mitosis, cytosolic supernumerary MTOCs are not likely to interfere with spindle formation in prometaphase and chromosome segregation. This implies that the mild mitotic defects leading to a slightly increased ploidy may result from a shortage of Cep192 at mitotic spindle poles, where Cep192 should be required for the binding of spindle microtubule nucleation complexes.

## 5. Conclusions

We have presented evidence that *Dictyostelium* Cep192 is the major component of the outer core layers and is required both for centrosome biogenesis and for integrity of the corona. Furthermore, superresolution microscopy revealed that our previous view with a subdivision of the *Dictyostelium* centrosome into a three-layered core structure surrounded by a corona was oversimplified. In fact, the corona should be subdivided into two distinct sheaths, one adjacent to the layered core and mainly consisting of CDK5RAP2, and another, distal sheath, containing the majority of the microtubule-nucleating corona proteins. Moreover, within the layered core structure, we should distinguish five layers, since both outer layers are flanked by a CP91 layer between them and the central layer. As electron microscopy shows only three major layers with a central layer that disappears during mitosis, we still count all core proteins disappearing during mitosis to the central layer and suggest a subdivision of the central layer into three sublayers.

## Data Availability

Genomic data on the *cepH* gene are available at https://dictybase.org, accessed on 7 September 2021. Further original data are available upon request from the corresponding author.

## Supplementary Materials

The following are available online at www.mdpi.com/article/10.3390/cells10092384/s1, Figure S1: Cep192 colocalizes at nuclear GFP-CDK5RAP2 foci (green) in cells overexpressing the GFP fusion protein. Staining with anti-’Cep192/anti-rabbit-AlexaFluor-568 (red) and anti-CP224/anti-mouse-AlexaFluor-633. CP224 staining labels the centrosome (not included in the merged image). Maximum intensity projections of deconvolved image stacks are shown. DNA is stained with DAPI (blue). Figure S2: Mix of Cep192-RNAi cells with GFP-α-Tubulin cells. Open arrow heads point at Cep192 signals in GFP-α-tubulin cells, filled arrow heads mark the signal in Cep192-RNAi cells. Cells were methanol fixed and stained with anti-Cep192/anti-rabbit-AlexaFluor-568. Maximum intensity projection of not further processed image. Bar = 10 nm. Video S1: GFP-Cep192 shows hardly any recovery after photobleaching (see Figure 1). Video S2: Cep192-GFP is present during the splitting of the mitotic centrosome (see Figure 2). Video S3: Live cell imaging of GFP-Cep192 during mitosis (see Figure 5).
