# Supplementary figures and images for: Cep192, a Novel Missing Link between the Centrosomal Core and Corona in Dictyostelium Amoebae
Source: Cells. 2021 Sep 10;10(9):2384. doi: 10.3390/cells10092384 (PMC8467581; doi:10.3390/cells10092384)

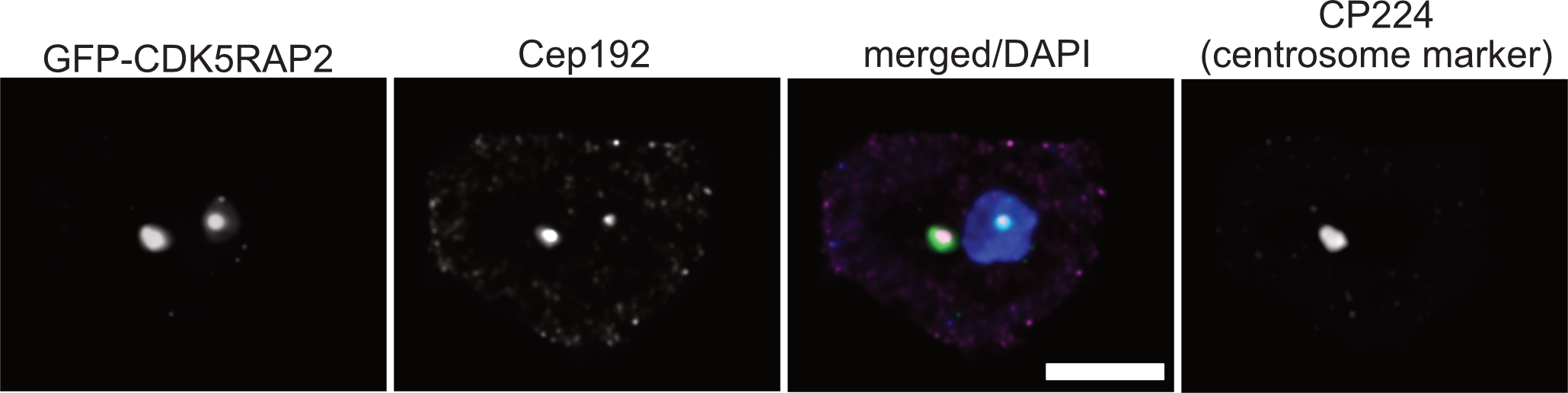

Supplement: Supplementary file 1 [file cells-10-02384-s001.zip › Figure S1.png]

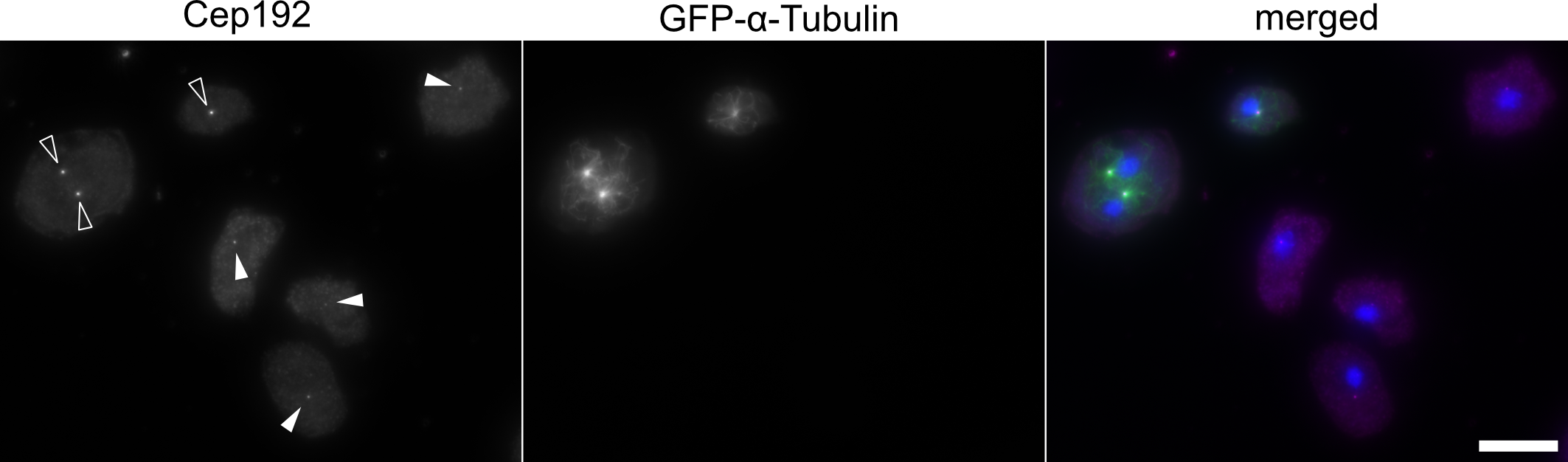

Supplement: Supplementary file 1 [file cells-10-02384-s001.zip › Figure S2.png]
